# (η^5^-Cyclo­penta­dien­yl)[(1,2,3,4,4a,12a-η)-naphtho­[2,3-*b*][1,4]benzodioxine]iron(II) hexa­fluoridophosphate

**DOI:** 10.1107/S1600536810033179

**Published:** 2010-08-21

**Authors:** Arthur D. Hendsbee, Jason D. Masuda, Adam Piórko

**Affiliations:** a Department of Chemistry, Saint Mary’s University, Halifax, Nova Scotia, Canada B3H 3C3

## Abstract

At 296 (2) K, both complexed rings in the iron(II) complex cation of the title salt, [Fe(C_5_H_5_)(C_16_H_10_O_2_)]PF_6_, are almost parallel [dihedral angle between planes = 2.4 (3)°]. The quaternary C atoms of the complexed arene ring are located at the longest distance from the Fe atom, with Fe—C distances of 2.112 (4) and 2.105 (3) Å, which are slightly longer than the average Fe—C distance for this ring (2.083 Å). The Fe ion is located 1.660 (1) and 1.543 (1) Å, respectively, from the cyclo­penta­dienyl and the complexed arene ring.

## Related literature

For the synthesis of the title compound and related structures, see Sutherland *et al.* (1982 ▶, 1988 ▶). For the crystal structures of similar polycyclic {(η^5^-Cp) (η^6^-arene) Fe(II)}^+^ salts, see Piórko *et al.* (1995 ▶); Benites *et al.* (1996 ▶, 1999 ▶); Decken (2004 ▶); Zanello *et al.* (2009 ▶) and literature cited therein; Asiri *et al.* (2010 ▶).
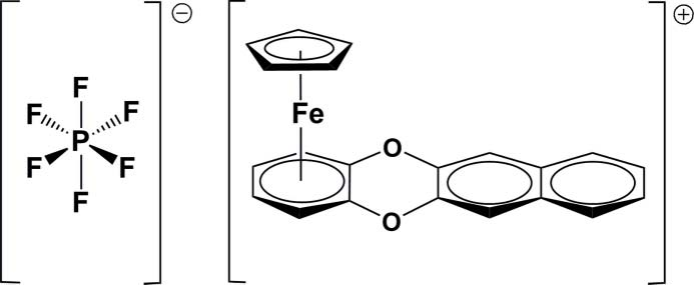


## Experimental

### 

#### Crystal data

[Fe(C_5_H_5_)(C_16_H_10_O_2_)]PF_6_
*M* *_r_* = 500.15
Monoclinic, 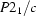

*a* = 15.3216 (13) Å
*b* = 8.9296 (8) Å
*c* = 14.6559 (12) Å
β = 106.417 (1)°
*V* = 1923.4 (3) Å^3^
*Z* = 4
Mo *K*α radiation
μ = 0.94 mm^−1^
*T* = 296 K
0.35 × 0.29 × 0.17 mm

#### Data collection

Bruker APEXII CCD diffractometer
Absorption correction: multi-scan (*SADABS*; Bruker, 2010 ▶) *T*_min_ = 0.576, *T*_max_ = 0.746
12307 measured reflections
3372 independent reflections
2360 reflections with *I* > 2σ(*I*)
*R*_int_ = 0.035

#### Refinement

*R*[*F* ^2^ > 2σ(*F* ^2^)] = 0.040
*wR*(*F* ^2^) = 0.115
*S* = 1.01
3372 reflections
318 parameters
10 restraints
H-atom parameters constrained
Δρ_max_ = 0.39 e Å^−3^
Δρ_min_ = −0.38 e Å^−3^

### 

Data collection: *APEX2* (Bruker, 2010 ▶); cell refinement: *SAINT* (Bruker, 2010 ▶); data reduction: *SAINT* ; program(s) used to solve structure: *SHELXS97* (Sheldrick, 2008 ▶); program(s) used to refine structure: *SHELXL97* (Sheldrick, 2008 ▶); molecular graphics: *ORTEP-3 for Windows* (Farrugia, 1997 ▶); software used to prepare material for publication: *SHELXL97*.

## Supplementary Material

Crystal structure: contains datablocks I, global. DOI: 10.1107/S1600536810033179/si2288sup1.cif

Structure factors: contains datablocks I. DOI: 10.1107/S1600536810033179/si2288Isup2.hkl

Additional supplementary materials:  crystallographic information; 3D view; checkCIF report

## Figures and Tables

**Fig. 1.**
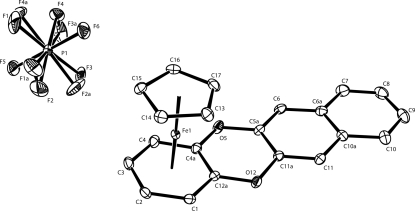
View of the complex showing the labelling of non-H atoms with the displacement ellipsoids shown at 50% probability levels.

## supplementary crystallographic information

### Comment

The title compound, along with similar polycyclic aromatic O-, S-, and N-containing heterocycles complexed with a cyclopentadienyliron(II) moiety, was reported from the study on nucleophilic aromatic di-substitution reactions using 1,2-dichlorobenzene FeCp complex (Sutherland *et al.*, 1988), which was an extension of an earlier study on the same reaction leading to synthesis of heterocyclic systems related to 9,10-dihydroanthracene and containing two heteroatoms at the 9,10-positions (Sutherland *et al.*, 1982).

The *ORTEP* of the title compound is shown in Figure 1. The planes of the coordinated arene ring and Cp ring are nearly parallel, with an angle of 2.4 (3)° between them, and this value is typically reported for benzodioxine–Fe–Cp complexes (see Piórko *et al.*, 1995, and references therein) and for arene–Fe–Cp complexes, in general (see for example Benites *et al.*, 1996; Benites *et al.*, 1999; Decken, 2004; Zanello *et al.*, 2009).

The Fe ion is located at the distances 1.660 (1)Å from the Cp ring and 1.543 (1)Å from the complexed arene ring, and these values are close to those reported in the literature for similar complexes (see for example Piórko *et al.*, 1995; Benites *et al.*, 1999; Decken, 2004, and literature cited therein).

In a complexed arene ring, the C–C bond lengths are found within the narrow range from 1.391 (6) to 1.409 (5) Å. Both oxygen atoms show similar bond lengths toward complexed arene ring carbon atoms [1.363 (4)Å and 1.362 (4) Å] and these appear to be shorter than similar bonds toward an uncomplexed ring [both at 1.389 (4) Å]. Similar trends have been reported for other dibenzodioxine complexes (see Piórko *et al.*, 1995). Of the C–C bonds in the uncomplexed fused carbocyclic rings of the heterocycle three appear to be markedly shorter [range 1.348 (6) to 1.356 (5) Å], one of intermediate length [1.373 (6) Å], and remaining seven appear to be longer [the range from 1.396 (7) to 1.423 (5) Å]. Some of the angles in the structure of a heterocycle appear to be distorted with angles C4a–O5–C5a and C11*a*–O12–C12*a* [116.7 (3) and 116.2 (3)°, respectively] and angles C6–C6a–C7 and C10–C10*a*–C11 [122.0 (4) and 122.2 (4)°, respectively, showing the largest deviations from an idealized trigonal geometry. The distribution of both the bond lengths and angles for the naphtho-moiety of this heterocycle are similar to the values reported for the naphthalene moiety of the naphthalene-2,3-diol in complex with 4-aminoantipyrine, with angles being less severely distorted from idealized geometry than those reported in the cited work (Asiri *et al.*, 2010).

### Experimental

The title complex was prepared following the procedure of Sutherland *et al.* (1988). A crystal used for data collection was grown by slow evaporation of solvents from a solution of the complex in acetone-diethyl ether-dichloromethane mixture at 280 K.

### Refinement

The H atoms were placed in geometrically idealized positions with C-H distances of 0.98Å  (complexed aromatic) and 0.93Å (aromatic). H atoms were constrained to ride on the parent C atom with Uiso(H) = 1.2Ueq(C) for aromatic and Uiso(H) = 1.2Ueq(C) for the idealized tertiary protons. The equatorial fluorines on the PF_6_ anion were modelled with a disorder ratio of 49 (2):51 (2) in order to obtain an adequate model. The P-F distances in the disordered PF_6_ anion were restrained to be within 1.55Å.

### Crystal data

**Table:**

[Fe(C_5_H_5_)(C_16_H_10_O_2_)]PF_6_	*F*(000) = 1008
*M**_r_* = 500.15	*D*_x_ = 1.727 Mg m^−^^3^
Monoclinic, *P*2_1_/*c*	Mo *K*α radiation, λ = 0.71073 Å
Hall symbol: -P 2ybc	Cell parameters from 2735 reflections
*a* = 15.3216 (13) Å	θ = 2.7–22.8°
*b* = 8.9296 (8) Å	µ = 0.94 mm^−^^1^
*c* = 14.6559 (12) Å	*T* = 296 K
β = 106.417 (1)°	Block, green
*V* = 1923.4 (3) Å^3^	0.35 × 0.29 × 0.17 mm
*Z* = 4	

### Data collection

**Table:**

Bruker APEXII CCD diffractometer	3372 independent reflections
Radiation source: fine-focus sealed tube	2360 reflections with *I* > 2σ(*I*)
graphite	*R*_int_ = 0.035
φ and ω scans	θ_max_ = 25.0°, θ_min_ = 2.7°
Absorption correction: multi-scan (*SADABS*; Bruker, 2010)	*h* = −18→18
*T*_min_ = 0.576, *T*_max_ = 0.746	*k* = −10→7
12307 measured reflections	*l* = −17→17

### Refinement

**Table:**

Refinement on *F*^2^	Primary atom site location: structure-invariant direct methods
Least-squares matrix: full	Secondary atom site location: difference Fourier map
*R*[*F*^2^ > 2σ(*F*^2^)] = 0.040	Hydrogen site location: inferred from neighbouring sites
*wR*(*F*^2^) = 0.115	H-atom parameters constrained
*S* = 1.01	*w* = 1/[σ^2^(*F*_o_^2^) + (0.0615*P*)^2^ + 0.6855*P*] where *P* = (*F*_o_^2^ + 2*F*_c_^2^)/3
3372 reflections	(Δ/σ)_max_ < 0.001
318 parameters	Δρ_max_ = 0.39 e Å^−^^3^
10 restraints	Δρ_min_ = −0.38 e Å^−^^3^

### Special details

**Table:**

Geometry. All e.s.d.'s (except the e.s.d. in the dihedral angle between two l.s. planes) are estimated using the full covariance matrix. The cell e.s.d.'s are taken into account individually in the estimation of e.s.d.'s in distances, angles and torsion angles; correlations between e.s.d.'s in cell parameters are only used when they are defined by crystal symmetry. An approximate (isotropic) treatment of cell e.s.d.'s is used for estimating e.s.d.'s involving l.s. planes.
Refinement. Refinement of *F*^2^ against ALL reflections. The weighted *R*-factor *wR* and goodness of fit *S* are based on *F*^2^, conventional *R*-factors *R* are based on *F*, with *F* set to zero for negative *F*^2^. The threshold expression of *F*^2^ > σ(*F*^2^) is used only for calculating *R*-factors(gt) *etc*. and is not relevant to the choice of reflections for refinement. *R*-factors based on *F*^2^ are statistically about twice as large as those based on *F*, and *R*- factors based on ALL data will be even larger.

### Fractional atomic coordinates and isotropic or equivalent isotropic displacement parameters (Å^2^)

**Table:**

	*x*	*y*	*z*	*U*_iso_*/*U*_eq_	Occ. (<1)
Fe1	0.16289 (3)	0.26572 (5)	0.26515 (3)	0.04229 (18)	
C1	0.1824 (3)	0.0364 (4)	0.2519 (3)	0.0590 (10)	
H1	0.1483	−0.0343	0.2799	0.071*	
C2	0.1398 (3)	0.0996 (5)	0.1626 (3)	0.0702 (12)	
H2	0.0772	0.0710	0.1293	0.084*	
C3	0.1777 (3)	0.2252 (5)	0.1324 (3)	0.0700 (12)	
H3	0.1408	0.2827	0.0782	0.084*	
C4	0.2580 (3)	0.2887 (5)	0.1898 (3)	0.0627 (11)	
H4	0.2763	0.3889	0.1752	0.075*	
C4A	0.3014 (2)	0.2225 (4)	0.2783 (2)	0.0524 (9)	
O5	0.37912 (17)	0.2877 (3)	0.33276 (19)	0.0654 (7)	
C11	0.4064 (2)	0.0586 (4)	0.5466 (2)	0.0507 (9)	
H11	0.3785	−0.0222	0.5670	0.061*	
C6	0.4885 (2)	0.2990 (4)	0.4823 (3)	0.0601 (10)	
H6	0.5157	0.3786	0.4598	0.072*	
C5A	0.4136 (2)	0.2335 (4)	0.4248 (3)	0.0507 (9)	
C6A	0.5258 (2)	0.2478 (4)	0.5763 (3)	0.0583 (10)	
C10A	0.4838 (2)	0.1264 (4)	0.6094 (2)	0.0534 (9)	
C10	0.5215 (3)	0.0752 (5)	0.7038 (3)	0.0711 (12)	
H10	0.4945	−0.0042	0.7268	0.085*	
C7	0.6054 (3)	0.3126 (6)	0.6385 (3)	0.0814 (14)	
H7	0.6340	0.3917	0.6173	0.098*	
C8	0.6396 (3)	0.2606 (6)	0.7278 (4)	0.0885 (15)	
H8	0.6918	0.3041	0.7675	0.106*	
C9	0.5979 (3)	0.1423 (7)	0.7616 (3)	0.0829 (14)	
H9	0.6220	0.1087	0.8237	0.099*	
O12	0.29927 (16)	0.0364 (3)	0.39696 (17)	0.0591 (7)	
C11A	0.3728 (2)	0.1115 (4)	0.4568 (2)	0.0474 (8)	
C12A	0.2627 (2)	0.0985 (4)	0.3096 (2)	0.0508 (9)	
C15	0.0678 (3)	0.4333 (4)	0.2353 (3)	0.0624 (10)	
H15	0.0418	0.4804	0.1731	0.075*	
C16	0.1460 (3)	0.4808 (4)	0.3056 (3)	0.0635 (10)	
H16	0.1842	0.5667	0.3010	0.076*	
C13	0.0896 (3)	0.2764 (5)	0.3621 (3)	0.0658 (11)	
H13	0.0812	0.1949	0.4036	0.079*	
C14	0.0335 (3)	0.3075 (5)	0.2704 (3)	0.0636 (10)	
H14	−0.0209	0.2509	0.2367	0.076*	
C17	0.1598 (3)	0.3829 (5)	0.3837 (3)	0.0673 (12)	
H17	0.2091	0.3891	0.4431	0.081*	
P1	0.14134 (7)	0.71338 (11)	0.01907 (6)	0.0534 (3)	
F6	0.1339 (2)	0.7270 (3)	0.12317 (14)	0.1027 (10)	
F5	0.1492 (2)	0.7032 (4)	−0.08442 (13)	0.1125 (11)	
F1	0.0591 (9)	0.8194 (19)	−0.0190 (13)	0.151 (7)	0.49 (2)
F4	0.2020 (9)	0.8540 (11)	0.0478 (10)	0.099 (4)	0.49 (2)
F3	0.2262 (9)	0.613 (2)	0.0594 (8)	0.119 (5)	0.49 (2)
F2	0.0843 (16)	0.5689 (16)	−0.0101 (14)	0.171 (7)	0.49 (2)
F4A	0.0861 (10)	0.8595 (11)	−0.0107 (10)	0.108 (4)	0.51 (2)
F3A	0.2313 (9)	0.802 (2)	0.0361 (11)	0.149 (6)	0.51 (2)
F2A	0.1942 (15)	0.5649 (16)	0.0482 (15)	0.165 (7)	0.51 (2)
F1A	0.0482 (7)	0.634 (2)	0.0012 (11)	0.119 (5)	0.51 (2)

### Atomic displacement parameters (Å^2^)

**Table:**

	*U*^11^	*U*^22^	*U*^33^	*U*^12^	*U*^13^	*U*^23^
Fe1	0.0490 (3)	0.0417 (3)	0.0325 (3)	0.0032 (2)	0.00559 (19)	−0.0020 (2)
C1	0.066 (2)	0.042 (2)	0.061 (2)	0.0015 (18)	0.0035 (19)	−0.0074 (18)
C2	0.081 (3)	0.066 (3)	0.049 (2)	0.014 (2)	−0.004 (2)	−0.020 (2)
C3	0.089 (3)	0.083 (3)	0.0362 (19)	0.023 (3)	0.013 (2)	−0.004 (2)
C4	0.077 (3)	0.069 (3)	0.051 (2)	0.016 (2)	0.031 (2)	0.0117 (19)
C4A	0.052 (2)	0.053 (2)	0.053 (2)	0.0079 (18)	0.0169 (17)	0.0044 (17)
O5	0.0552 (15)	0.0659 (19)	0.0713 (17)	−0.0065 (13)	0.0116 (13)	0.0257 (14)
C11	0.0431 (19)	0.051 (2)	0.059 (2)	0.0058 (17)	0.0171 (17)	0.0088 (17)
C6	0.050 (2)	0.043 (2)	0.083 (3)	−0.0032 (17)	0.011 (2)	0.0063 (19)
C5A	0.0469 (19)	0.043 (2)	0.062 (2)	0.0027 (16)	0.0143 (17)	0.0099 (17)
C6A	0.045 (2)	0.052 (3)	0.073 (3)	0.0043 (18)	0.0101 (18)	−0.0073 (19)
C10A	0.0417 (19)	0.063 (3)	0.053 (2)	0.0115 (17)	0.0093 (17)	−0.0078 (18)
C10	0.059 (2)	0.097 (4)	0.058 (2)	0.014 (2)	0.017 (2)	0.000 (2)
C7	0.060 (3)	0.072 (3)	0.096 (4)	−0.004 (2)	−0.004 (2)	−0.010 (3)
C8	0.067 (3)	0.097 (4)	0.085 (3)	0.001 (3)	−0.005 (3)	−0.026 (3)
C9	0.063 (3)	0.119 (4)	0.058 (3)	0.017 (3)	0.003 (2)	−0.014 (3)
O12	0.0536 (14)	0.0509 (16)	0.0604 (15)	−0.0074 (12)	−0.0039 (12)	0.0155 (12)
C11A	0.0402 (18)	0.045 (2)	0.054 (2)	0.0021 (15)	0.0078 (16)	0.0021 (16)
C12A	0.056 (2)	0.041 (2)	0.052 (2)	0.0061 (17)	0.0087 (17)	0.0007 (16)
C15	0.060 (2)	0.055 (3)	0.069 (2)	0.019 (2)	0.014 (2)	0.007 (2)
C16	0.071 (3)	0.045 (2)	0.080 (3)	0.001 (2)	0.030 (2)	−0.017 (2)
C13	0.074 (3)	0.079 (3)	0.051 (2)	−0.002 (2)	0.029 (2)	−0.003 (2)
C14	0.052 (2)	0.066 (3)	0.072 (3)	0.000 (2)	0.014 (2)	−0.007 (2)
C17	0.074 (3)	0.079 (3)	0.047 (2)	0.006 (2)	0.0152 (19)	−0.020 (2)
P1	0.0587 (6)	0.0512 (6)	0.0445 (5)	0.0020 (5)	0.0052 (4)	0.0036 (4)
F6	0.118 (2)	0.131 (3)	0.0626 (16)	0.0151 (18)	0.0325 (16)	0.0094 (15)
F5	0.163 (3)	0.120 (3)	0.0552 (15)	0.042 (2)	0.0322 (17)	0.0069 (15)
F1	0.066 (6)	0.242 (18)	0.118 (8)	0.079 (8)	−0.017 (5)	0.026 (11)
F4	0.137 (9)	0.065 (5)	0.092 (5)	−0.039 (5)	0.030 (7)	−0.004 (4)
F3	0.133 (8)	0.154 (10)	0.059 (5)	0.108 (8)	0.009 (5)	0.012 (6)
F2	0.236 (14)	0.096 (9)	0.143 (10)	−0.088 (10)	−0.011 (12)	−0.016 (7)
F4A	0.168 (12)	0.062 (5)	0.103 (8)	0.041 (5)	0.051 (8)	0.021 (4)
F3A	0.067 (5)	0.273 (18)	0.095 (7)	−0.068 (8)	0.004 (4)	0.017 (9)
F2A	0.256 (15)	0.100 (8)	0.171 (15)	0.100 (9)	0.115 (12)	0.081 (8)
F1A	0.101 (6)	0.137 (10)	0.123 (6)	−0.074 (7)	0.036 (5)	−0.029 (8)

### Geometric parameters (Å, °)

**Table:**

Fe1—C17	2.040 (4)	C5A—C11A	1.402 (5)
Fe1—C14	2.040 (4)	C6A—C10A	1.414 (5)
Fe1—C15	2.048 (4)	C6A—C7	1.423 (5)
Fe1—C13	2.047 (4)	C10A—C10	1.417 (5)
Fe1—C16	2.048 (4)	C10—C9	1.373 (6)
Fe1—C3	2.053 (4)	C10—H10	0.9300
Fe1—C2	2.070 (4)	C7—C8	1.348 (6)
Fe1—C4	2.072 (4)	C7—H7	0.9300
Fe1—C1	2.086 (4)	C8—C9	1.396 (7)
Fe1—C12A	2.105 (3)	C8—H8	0.9300
Fe1—C4A	2.112 (4)	C9—H9	0.9300
C1—C12A	1.396 (5)	O12—C12A	1.362 (4)
C1—C2	1.406 (5)	O12—C11A	1.389 (4)
C1—H1	0.9800	C15—C14	1.398 (5)
C2—C3	1.391 (6)	C15—C16	1.407 (5)
C2—H2	0.9800	C15—H15	0.9800
C3—C4	1.400 (6)	C16—C17	1.407 (6)
C3—H3	0.9800	C16—H16	0.9800
C4—C4A	1.409 (5)	C13—C14	1.403 (5)
C4—H4	0.9800	C13—C17	1.403 (5)
C4A—O5	1.363 (4)	C13—H13	0.9800
C4A—C12A	1.394 (5)	C14—H14	0.9800
O5—C5A	1.389 (4)	C17—H17	0.9800
C11—C11A	1.356 (5)	P1—F4	1.549 (2)
C11—C10A	1.415 (5)	P1—F1	1.548 (2)
C11—H11	0.9300	P1—F2	1.549 (2)
C6—C5A	1.350 (5)	P1—F3	1.550 (2)
C6—C6A	1.410 (5)	P1—F5	1.5575 (16)
C6—H6	0.9300	P1—F6	1.5664 (17)
			
C17—Fe1—C14	67.49 (16)	C4—C4A—Fe1	68.8 (2)
C17—Fe1—C15	67.71 (16)	C4A—O5—C5A	116.7 (3)
C14—Fe1—C15	40.00 (15)	C11A—C11—C10A	120.0 (3)
C17—Fe1—C13	40.16 (15)	C11A—C11—H11	120.0
C14—Fe1—C13	40.16 (15)	C10A—C11—H11	120.0
C15—Fe1—C13	67.51 (17)	C5A—C6—C6A	120.5 (4)
C17—Fe1—C16	40.26 (16)	C5A—C6—H6	119.8
C14—Fe1—C16	67.26 (17)	C6A—C6—H6	119.8
C15—Fe1—C16	40.18 (15)	C6—C5A—O5	118.6 (3)
C13—Fe1—C16	67.41 (17)	C6—C5A—C11A	120.6 (3)
C17—Fe1—C3	158.83 (19)	O5—C5A—C11A	120.8 (3)
C14—Fe1—C3	115.88 (17)	C6—C6A—C10A	119.1 (3)
C15—Fe1—C3	100.87 (17)	C6—C6A—C7	122.0 (4)
C13—Fe1—C3	153.49 (18)	C10A—C6A—C7	118.9 (4)
C16—Fe1—C3	119.78 (18)	C11—C10A—C6A	118.9 (3)
C17—Fe1—C2	161.04 (19)	C11—C10A—C10	122.2 (4)
C14—Fe1—C2	100.97 (17)	C6A—C10A—C10	118.9 (4)
C15—Fe1—C2	113.79 (16)	C9—C10—C10A	120.2 (5)
C13—Fe1—C2	121.53 (18)	C9—C10—H10	119.9
C16—Fe1—C2	150.78 (17)	C10A—C10—H10	119.9
C3—Fe1—C2	39.42 (16)	C8—C7—C6A	120.7 (5)
C17—Fe1—C4	126.30 (18)	C8—C7—H7	119.7
C14—Fe1—C4	147.06 (16)	C6A—C7—H7	119.7
C15—Fe1—C4	112.27 (16)	C7—C8—C9	120.9 (4)
C13—Fe1—C4	166.28 (17)	C7—C8—H8	119.6
C16—Fe1—C4	103.14 (17)	C9—C8—H8	119.6
C3—Fe1—C4	39.66 (17)	C10—C9—C8	120.5 (4)
C2—Fe1—C4	71.66 (18)	C10—C9—H9	119.7
C17—Fe1—C1	128.39 (16)	C8—C9—H9	119.7
C14—Fe1—C1	110.46 (17)	C12A—O12—C11A	116.2 (3)
C15—Fe1—C1	144.04 (16)	C11—C11A—O12	117.5 (3)
C13—Fe1—C1	103.38 (17)	C11—C11A—C5A	120.9 (3)
C16—Fe1—C1	168.65 (15)	O12—C11A—C5A	121.5 (3)
C3—Fe1—C1	71.46 (17)	O12—C12A—C4A	122.2 (3)
C2—Fe1—C1	39.54 (14)	O12—C12A—C1	117.8 (3)
C4—Fe1—C1	84.65 (17)	C4A—C12A—C1	120.0 (3)
C17—Fe1—C12A	106.86 (15)	O12—C12A—Fe1	130.5 (2)
C14—Fe1—C12A	138.34 (16)	C4A—C12A—Fe1	71.0 (2)
C15—Fe1—C12A	174.54 (14)	C1—C12A—Fe1	69.8 (2)
C13—Fe1—C12A	108.15 (16)	C14—C15—C16	107.6 (4)
C16—Fe1—C12A	135.72 (15)	C14—C15—Fe1	69.7 (2)
C3—Fe1—C12A	84.45 (15)	C16—C15—Fe1	69.9 (2)
C2—Fe1—C12A	71.12 (14)	C14—C15—H15	126.2
C4—Fe1—C12A	71.10 (14)	C16—C15—H15	126.2
C1—Fe1—C12A	38.90 (13)	Fe1—C15—H15	126.2
C17—Fe1—C4A	106.32 (15)	C15—C16—C17	108.1 (4)
C14—Fe1—C4A	172.93 (14)	C15—C16—Fe1	69.9 (2)
C15—Fe1—C4A	142.10 (16)	C17—C16—Fe1	69.6 (2)
C13—Fe1—C4A	132.83 (15)	C15—C16—H16	126.0
C16—Fe1—C4A	110.57 (15)	C17—C16—H16	126.0
C3—Fe1—C4A	71.14 (16)	Fe1—C16—H16	126.0
C2—Fe1—C4A	84.02 (16)	C14—C13—C17	107.7 (4)
C4—Fe1—C4A	39.33 (14)	C14—C13—Fe1	69.6 (2)
C1—Fe1—C4A	70.26 (15)	C17—C13—Fe1	69.6 (2)
C12A—Fe1—C4A	38.60 (13)	C14—C13—H13	126.1
C12A—C1—C2	120.2 (4)	C17—C13—H13	126.1
C12A—C1—Fe1	71.3 (2)	Fe1—C13—H13	126.1
C2—C1—Fe1	69.6 (2)	C15—C14—C13	108.6 (4)
C12A—C1—H1	119.1	C15—C14—Fe1	70.3 (2)
C2—C1—H1	119.1	C13—C14—Fe1	70.2 (2)
Fe1—C1—H1	119.1	C15—C14—H14	125.7
C3—C2—C1	119.6 (4)	C13—C14—H14	125.7
C3—C2—Fe1	69.6 (2)	Fe1—C14—H14	125.7
C1—C2—Fe1	70.9 (2)	C13—C17—C16	108.0 (4)
C3—C2—H2	119.3	C13—C17—Fe1	70.2 (2)
C1—C2—H2	119.3	C16—C17—Fe1	70.2 (2)
Fe1—C2—H2	119.3	C13—C17—H17	126.0
C2—C3—C4	120.7 (4)	C16—C17—H17	126.0
C2—C3—Fe1	70.9 (2)	Fe1—C17—H17	126.0
C4—C3—Fe1	70.9 (2)	F4—P1—F1	88.1 (7)
C2—C3—H3	118.9	F4—P1—F2	177.5 (9)
C4—C3—H3	118.9	F1—P1—F2	94.1 (9)
Fe1—C3—H3	118.9	F4—P1—F3	89.4 (7)
C3—C4—C4A	119.3 (4)	F1—P1—F3	177.4 (8)
C3—C4—Fe1	69.5 (2)	F2—P1—F3	88.4 (9)
C4A—C4—Fe1	71.9 (2)	F4—P1—F5	96.1 (5)
C3—C4—H4	119.7	F1—P1—F5	87.9 (7)
C4A—C4—H4	119.7	F2—P1—F5	82.8 (9)
Fe1—C4—H4	119.7	F3—P1—F5	92.9 (5)
O5—C4A—C12A	121.9 (3)	F4—P1—F6	82.8 (5)
O5—C4A—C4	117.8 (3)	F1—P1—F6	91.6 (7)
C12A—C4A—C4	120.1 (4)	F2—P1—F6	98.2 (9)
O5—C4A—Fe1	131.6 (3)	F3—P1—F6	87.5 (5)
C12A—C4A—Fe1	70.4 (2)	F5—P1—F6	178.9 (2)
